# Mutation Breeding of Extracellular Polysaccharide-Producing Microalga *Crypthecodinium cohnii* by a Novel Mutagenesis with Atmospheric and Room Temperature Plasma

**DOI:** 10.3390/ijms16048201

**Published:** 2015-04-13

**Authors:** Bin Liu, Zheng Sun, Xiaonian Ma, Bo Yang, Yue Jiang, Dong Wei, Feng Chen

**Affiliations:** 1 School of Light Industry and Food Sciences, South China University of Technology, Guangzhou 510641, China; 2 Institute for Food and Bioresource Engineering, College of Engineering, Peking University, Beijing 100871, China; 3 College of Fisheries and Life Science, Shanghai Ocean University, Shanghai 201306, China; 4 School of Food Science, Jiangnan University, Wuxi 214122, China; 5 Singapore-Peking University Research Centre for a Sustainable Low-Carbon Future, CREATE Tower, Singapore 138602, Singapore

**Keywords:** extracellular polysaccharides, atmospheric and room temperature plasma (ARTP), mutation, microalgae, *Crypthecodinium cohnii*

## Abstract

Extracellular polysaccharides (EPS) produced by marine microalgae have the potential to be used as antioxidants, antiviral agents, immunomodulators, and anti-inflammatory agents. Although the marine microalga *Crypthecodinium cohnii* releases EPS during the process of docosahexaenoic acid (DHA) production, the yield of EPS remains relatively low. To improve the EPS production, a novel mutagenesis of *C. cohnii* was conducted by atmospheric and room temperature plasma (ARTP). Of the 12 mutants obtained, 10 mutants exhibited significantly enhanced EPS yield on biomass as compared with the wild type strain. Among them, mutant M7 was the best as it could produce an EPS volumetric yield of 1.02 g/L, EPS yield on biomass of 0.39 g/g and EPS yield on glucose of 94 mg/g, which were 33.85%, 85.35% and 57.17% higher than that of the wild type strain, respectively. Results of the present study indicated that mutagenesis of the marine microalga *C. cohnii* by ARTP was highly effective leading to the high-yield production of EPS.

## 1. Introduction

Polysaccharides are polymeric carbohydrate molecules consisting of long chains of monosaccharide units. As a major class of biological macromolecules, they play important roles in a vast number of biochemical activities [1]. With the development of glycomics, the significant contribution of polysaccharides in human health has received increasing attention in recent years. There are a growing number of studies demonstrating the bio-modulatory effects of polysaccharides, e.g., anti-oxidative, anti-inflammatory, anti-adhesive, anti-coagulant, anti-cancer, anti-viral, and immunomodulatory properties [2,3,4]. Marine algae are known as one of the most abundant sources of polysaccharides: for example, Chrysophyta [5], *Phaeodactylum tricornutum* [6], *Nannochloropsis oculata* [7], *Porphyridium cruentum* [8,9], and *Gyrodinium impudium* [10] have been reported as good producers, and the yielded polysaccharides included alginates, carrageenans and agarose [2]. More importantly, the production of polysaccharides from marine microalgae is practically advantageous because the process is easily controlled and the harvesting does not depend on the climate or season [11].

Generally, polysaccharides from marine microalgae can be divided into structural polysaccharides, energy polysaccharides and extracellular polysaccharides based on their physiological roles [12]. From the commercialization point of view, extracellular polysaccharides (EPS) are the most promising ones because they can be diffused into the culture medium, free from cell debris, and easy to collect and purify [2,13]. In the present work, we used a novel mutagenesis tool named atmospheric and room temperature plasma (ARTP) to obtain algal strains with enhanced EPS content. The ARTP technique uses the helium radio-frequency glow discharge plasma jets to change the microbial DNA sequences, which possesses a number advantages over most traditional mutation systems [14]. In 2013 and 2014, ARTP has been successfully utilized for microbial breeding such as bacteria, fungi, and the cyanobactrium *Spirulina platensis* [14,15,16].

In this study, we applied it to the mutagenesis of eukaryotic microalgae. The strain we examined was the dinoflagellate microalga *Crypthecodinium cohnii*. *C. cohnii* can secrets EPS which have a great application potential in functional foods and pharmaceuticals. Although the EPS contentof *C. cohnii* is less than seaweeds, a type of well-known EPS producers, it is higher than most microalgae [17,18,19]. Besides, this strain is known as a prolific producer of docosahexaenoic acid (DHA), a high-value omega-3 polyunsaturated fatty acids possessing various physiological and nutritional functions [20]. This unique characteristic makes *C. cohnii* an advantageous candidate for the integrated utilization of EPS and high-value components will significantly bring down the cost of production. The aim of the present study was to investigate the possibility of using ARTP for the mutagenesis of *C. cohnii* and evaluate the EPS production potential of the selected *C. cohnii* mutants.

## 2. Results

### 2.1. Determination of the Optimal Sampling Time

To find the best sampling time, *C. cohnii* that grew after 24, 36, and 72 h were sampled to perform the ARTP treatment, which were corresponding to the beginning of log phase, the end of log phase, and the end of stationary phase, respectively.

**Figure 1:**
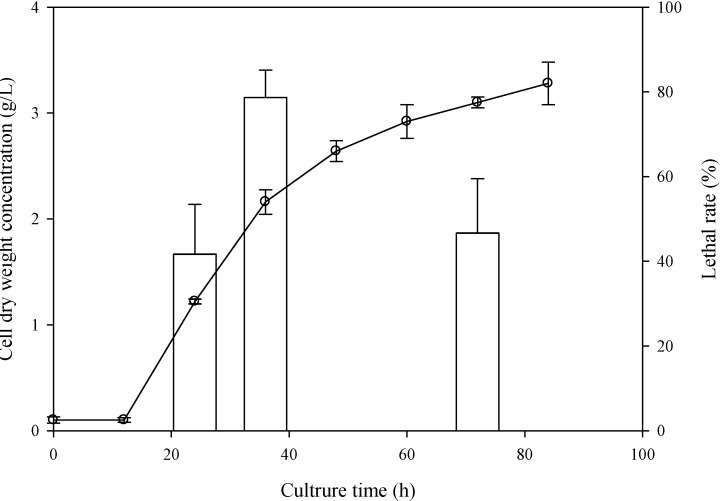
Effect of sampling time on the lethal rate of *C. cohnii* irradiated by atmospheric and room temperature plasma (ARTP): the line was the growth curve of *C. cohnii* for the mutagenesis; the columns were the lethal rate of *C. cohnii* sampled at 24, 36, and 72 h treated by ARTP with a radio-frequency (RF) power input of 150 W for 90 s.

As shown in Figure 1, the lethal rate of cells sampled at 36 h reached 78.68% after irradiating 90 s by APTP, which was much higher than those sampled at 24 and 72 h, indicating that *C. cohnii* sampled at 36 h was more sensitive to the plasma. Therefore, the plasma of ARTP system may easily lead to a lethal, cell permeability change or DNA damage of *C. cohnii* at the end of exponential phase.

### 2.2. Determination of the Optimal ARTP Treatment Duration

**Figure 2:**
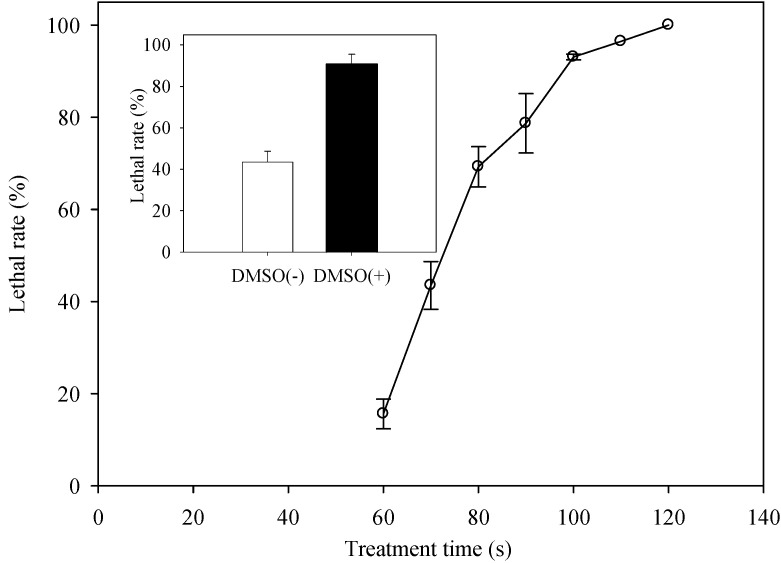
The relationship between treatment conditions of ARTP and lethal rate of *C. cohnii*. The curve representing lethal rate against treatment time; the white column: the lethal rate of cells diluted by By+ medium without dimethylsulfoxide (DMSO) treated for 70 s; the black column: the lethal rate of cells diluted by By+ medium with DMSO treated for 70 s; the irradiation conditions: *C. cohnii* sampled at 36 h, RF power input of 150 W, helium gas flow rate of 10 L/min, distance between the *C. cohnii* cells and the plasma source of 2 mm.

In order to obtain mutants with enhanced EPS content, the lethal rate should be taken into account. The curve of lethal rate against treatment duration is shown in Figure 2. The lethal rate was over 90% when the exposure time exceeded 100 s. There was no cell survival after treating for 120 s. Although it could meet the recommended lethal rate of 90%–99% after treating for 100 s, the cell suspension was almost dried up because of the exposure to the helium plasma flow.

Therefore, in order to protect cells from damage caused by water evaporation and increase the cell penetrability of plasma, DMSO, a reagent to enhance the cell membrane permeability, was mixed in the suspension [21]. In the presence of DMSO, the lethal rate reached to 90.74% when treated under the same conditions for 70 s (black bar in Figure 2). In contrast, the lethal rate was only 43.49% when exposed to the plasma for 70 s without DMSO (white bar in Figure 2). Taken together, *C. cohnii* sampled at 36 h were suspended in By+ medium with 5% DMSO and used for mutation by ARTP.

### 2.3. Screening for Mutants with Enhanced EPS Content

**Figure 3:**
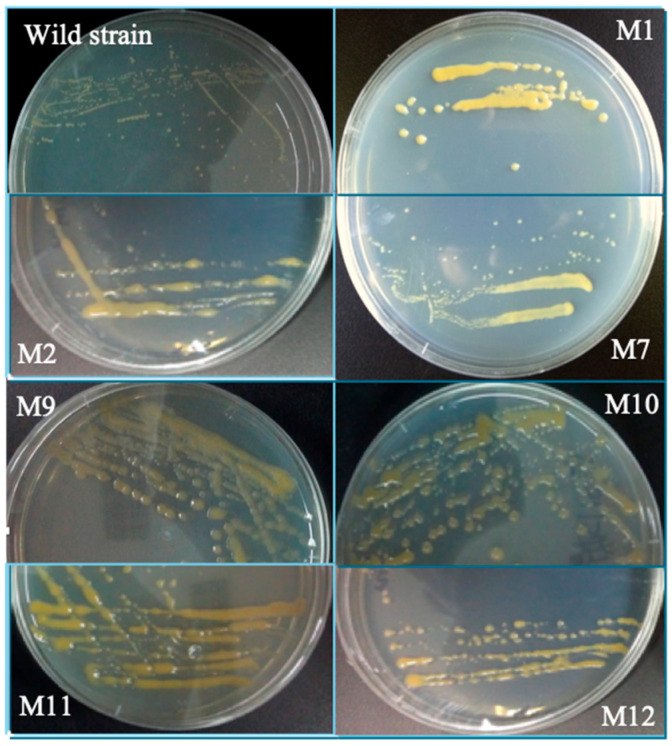
Colonies of wild type of *C. cohnii* and the mutants: the wild type and selected mutants of *C. cohnii* were grown on solid agar plates and incubated for 14 days.

After the treatment by ARTP, colonies of 12 mutants of *C. cohnii*, named M1 to M12, with smooth and moist surface were picked out and transferred to fresh agar plates. Compared with the wild type, the mutants formed larger colonies and most of them secreted more EPS (Figure 3).

### 2.4. EPS Production of the Mutants

**Figure 4:**
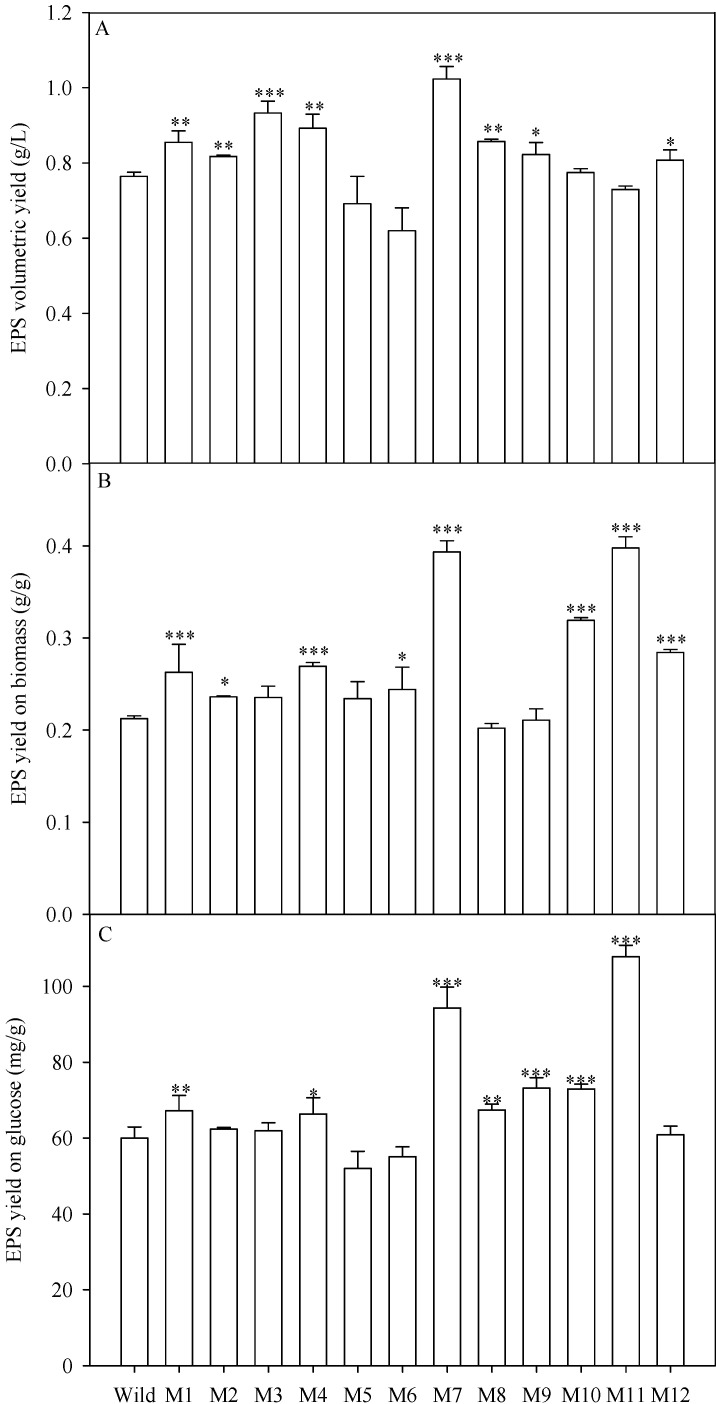
EPS yield of the wild type and 12 mutants of *C. cohnii*. (**A**) EPS volumetric yield; (**B**) EPS yield on biomass; (**C**) EPS yield on glucose; asterisk (* *p* < 0.05, ** *p* < 0.01, and *** *p* < 0.001) means significant differences between wild type and the mutants.

The wild type strain and the 12 mutants of *C. cohnii* were cultured in the modified By+ medium. After 96 h, the crude EPS in the culture were precipitated by ethanol and then lyophilized. The volumetric yield of EPS by 8 mutants (M1, M2, M3, M4, M7, M8, M9 and M12) increased significantly as compared with the wild type (Figure 4A). Mutant M7 produced the highest EPS volumetric yield of 1.02 g/L with an increase of 33.85%. The EPS volumetric yield of M3 was the second highest among all mutants with an increase of 22.03%.

The EPS yield based on biomass was also calculated to evaluate the EPS production ability. Results revealed that 10 mutants produced higher EPS yield on biomass (Figure 4B). Mutant M7 and M11 produced the highest EPS yield based on biomass. Nevertheless, M11 was not suitable for EPS production because its EPS volumetric yield was very low due to its low biomass concentration (Table 1).

**Table 1:** The cell dry weight concentration and growth yield on glucose of the wild type and 12 mutants of *C. cohnii*.

Species	Cell Dry Weight Concentration (g/L)	Growth Yield on Glucose (g/g)
wild type	3.60 ± 0.09	0.28 ± 0.02
M1	3.25 ± 0.29	0.26 ± 0.02
M2	3.46 ± 0.22	0.26 ± 0.00
M3	3.96 ± 0.32 ***	0.27 ± 0.01
M4	3.31 ± 0.10	0.25 ± 0.02
M5	2.95 ± 0.11	0.22 ± 0.01
M6	2.54 ± 0.02	0.23 ± 0.01
M7	2.60 ± 0.02	0.24 ± 0.01
M8	4.24 ± 0.09 ***	0.33 ± 0.00 ***
M9	3.90 ± 0.12 **	0.35 ± 0.02 ***
M10	2.43 ± 0.05	0.23 ± 0.01
M11	1.83 ± 0.08	0.27 ± 0.02
M12	2.84 ± 0.07	0.21 ± 0.01

Asterisk (** *p* < 0.01 and *** *p* < 0.001) means that there were significant differences between the wild type and the mutants.

Considering the consumption of glucose, the EPS yield on glucose of 7 mutants was enhanced as compared with the wild type (Figure 4C). M7 produced higher EPS yield on glucose of 94.3 mg/g with an increase of 57.17%. Although M11 produced significantly higher EPS yield on glucose (*i.e.*, 107.8 mg/g, with an increase of 79.67%) than M7 (*p* < 0.01), M11 only produced 71.29% EPS volumetric yield of M7.

Taken together, among all mutants, mutant M7 was most suitable for EPS production because of its highest EPS volumetric yield. The EPS volumetric yield, EPS yield on biomass and EPS yield on glucose of M7 were improved by 33.85%, 85.35% and 57.17%, respectively (Figure 4). Besides, M1 and M4 increased significantly in terms of EPS yield on biomass, EPS volumetric yield and EPS yield on glucose.

### 2.5. Monosaccharide Composition

The composition of EPS produced by the wild type and M7 was characterized (Table 2). Glucose and galactose turned out to be the predominant monosaccharides in the EPS of the wild type (*C. cohnii* ATCC 30556). Other sugars such as mannose, ribose and fucose, were also detected in the crude EPS (Table 2).

Monosaccharide composition of the EPS was also analyzed, and there was no significant difference between the wild type and M7 in monosaccharide composition (Table 2). These results indicated that mutant M7 secreted larger amounts of EPS whereas the monosaccharide composition remained unchanged as compared with the wild type.

**Table 2:** Monosaccharide composition (in mol %) of crude EPS from the wild type and mutant M7 of *C. cohnii*.

Species	Mannose	Ribose	Glucose	Galactose	Fucose	Unknown
Wild	6.53 ± 0.15	5.99 ± 0.51	61.73 ± 0.59	20.15 ± 0.06	3.48 ± 0.08	2.13 ± 0.05
M7	6.05 ± 0.01	4.72 ± 0.12	63.77 ± 0.12	19.61 ± 0.07	3.65 ± 0.28	2.2 ± 0.04

## 3. Discussion

ARTP is a novel, high-efficient and environment-friendly mutagenesis tool as compared with transgenic techniques or traditional chemical and physical mutagens [14]. The plasma from ARTP contains significant amounts of active chemical species which can improve cell permeability, penetrate the cell wall/membrane and cause DNA/protein damage [22,23]. In contrast to the traditional mutagens, the plasma has higher positive genotoxic response [15]. Besides, the plasma shows different genotoxic characteristics depending on the irradiation dose [15]. In the present investigation, the ARTP was conducted for the mutagenesis of eukaryotic microalga *C. cohnii*.

For the purpose of optimizing mutation conditions, the irradiation dose of ARTP should be taken into account. A lethal rate between 90%–99% favors the generation of mutants with higher EPS contents [14]. In the present study, the RF input power was set at 150 W, higher than the power of 100 W that was normally used [14,15,16]. We found the higher RF input power provided the plasma with high energy, which allows the plasma to efficiently penetrate through the cell wall of *C. cohnii* and thereby alert the genome.

The plasma also shows different genotoxic characteristics depending on the cultivation phase of the wild type strain. In the present study, *C. cohnii* cells in log phase were found to be most sensitive to the plasma. These results were consistent with previous reports suggesting cells grown at log phase are more suitable for mutation [16]. Although *C. cohnii* ATCC 30772 produced 2.7 g/L crude EPS, the EPS yield based on biomass was only 0.15 g/g [18]. For comparison, EPS yields on biomass for the marine microalgae *Aurantiochytrium*, *Schizochytrium*, *Ulkenia*, and *Thraustochytrium* (from 0.015 to 0.119 g/g) were even lower than *C. cohnii* [17]. In contrast, the EPS yield on biomass of mutant M7 of *C. cohnii* (ATCC 30556) could reach to 0.39 g/g which was 160% higher than that of *C. cohnii* ATCC 30772.

The genetic stability of mutant M7 was also investigated. After a 9-genertation culture, M7 still maintained high EPS yield on biomass of 0.38 ± 0.01 g/g. Although further research is necessary to explain the genome mutation mechanism by ARTP mutagenesis, the target products-producing ability of mutants also remained very stable after more than ten generations [16,24].

Among enzymes involved in the biosynthesis of EPS, alpha-phosphoglucomutase (PMG) and UDP-glucose pyrophosphorylase (UGP) play key roles, whose activities are highly correlated with the EPS level [25]. Overexpression of *PGM* gene leads to high production of EPS with higher transcription levels of *UGP* gene [26]. In future, the contribution of these two enzymes towards *C. cohnii* mutant needs to be determined, which may help elucidate the underlying mechanism of ARTP for improving EPS production of *C. cohnii*.

## 4. Experimental Section

### 4.1. Algal Species and Culture Conditions

The alga *Crypthecodinium cohnii* (ATCC 30556) was obtained from the American Type Culture Collection (Rockville, MD, USA). The strain was heterotrophically maintained in By+ medium (containing 15 g sea salt, 1 g yeast extract, 1 g tryptone, and 5 g glucose per liter) at 16 °C and subcultured every month. The inocula were prepared by culturing the alga in 500-mL Erlenmeyer flasks containing 100 mL By+ medium at 25 °C for 48 h with orbital shaking at 150 rpm in the dark. The seeds of wild type and mutants were inoculated on to 100 mL fresh modified By+ medium (containing 15 g sea salt, 0.5 g yeast extract, 0.5 g tryptone, and 20 g glucose per liter) at a 5% (*v*/*v*) inoculum size for batch culture in 500-mL Erlenmeyer flasks. The pH of the modified By+ medium was adjusted to 6.5 prior to autoclaving. After inoculating, flasks were incubated at 25 °C in an orbital shaker at 150 rpm in the dark for 96 h.

### 4.2. Mutation by ARTP

The artificial plasma used in the present mutation was generated by the helium-based ARTP mutation system (Si Qing Yuan Biotechnology Co., Ltd., Wuxi, China). About 50,000 cells at the end of log phase were suspended in 20 μL By+ medium (with 5% dimethylsulfoxide (DMSO)) and placed on a stainless steel minidisc. The plate was then exposed to the plasma and the operating parameters of ARTP system were set as follows: radio-frequency (RF) power input of 150 W, the helium gas flow rate of 10 L/min, treating distance of 2 mm, and the treating time ranged from 60 to 120 s. The control was treated for 120 s with the same parameters except no RF power input. After being treated by the artificial plasma for various time intervals, the *C. cohnii* samples were moved into By+ medium and diluted to an appropriate concentration. Cells were transferred on to a By+ solid medium and cultured at 25 °C in the dark for two weeks. The strategies for screening high-EPS mutants were based on the moisture, smoothness, and size of the colony growing on the solid plates. The lethal rate was calculated as follows: lethal rate = (1 − *N*_1_/*N*_0_) × 100%, where *N*_0_ is colony number of the control and *N*_1_ is colony number of mutation group growing on the plates. All treatments were determined in triplicates.

### 4.3. Isolation of Extracellular Polysaccharides

Cultures were centrifuged at 4000× *g* for 10 min. Two volumes of absolute ethanol were added to the supernatant to precipitate the EPS. After 6 h of treatment at room temperature (25 °C), the precipitation was harvested by centrifuging at 1000× *g* for 5 min. After being washed twice by 66% (*v*/*v*) ethanol in water, the precipitated crude EPS was redissolved in ddH_2_O and lyophilized [27]. The EPS production was determined by weighing the lyophilized crude EPS.

### 4.4. Analysis of Monosaccharide Composition

The crude EPS (0.5 mg) was hydrolyzed at 110 °C in 200 μL of 2 M trifluoroacetic acid (TFA) solution for 2 h. After cooling to room temperature, 200 μL of methanol was added to the hydrolysate which was then dried under N_2_, and this procedure was repeated twice to remove TFA. The hydrolyzed sugars were dissolved in 100 μL of 0.3 M NaOH solutions and mixed with 100 μL of 0.5 M 3-methyl-1-phenyl-2-pyrazoline-5-one (PMP) in methanol to derive the monosaccharide at 70 °C for 100 min. The reaction solution was then cooled to room temperature whose pH was adjusted to 7.0 by 0.3 M HCl solution and the volume was adjusted to 1 mL by ddH_2_O. Finally, the derived monosaccharide solution was filtered (with a 0.22 μm membrane) for high performance liquid chromatography (HPLC) analysis. The derived monosaccharide was measured by HPLC (2695, Waters Corporation, Milford, MA, USA) at 40 °C using a C18 column (4.6 mm × 250 mm, Waters, USA) and photo-diode array (PDA) detector. Phosphate buffer (0.1 M, pH 6.7, 83%) and acetonitrile (17%) were used as mobile phases with the flow rate at 1.0 mL/min. Five pure sugars including mannose, ribose, glucose, galactose, and fucose (purchased from Sigma-Aldrich, St. Louis, MO, USA), were used as the standards [28].

### 4.5. Determination of Residual Glucose Concentration and Cell Dry Weight

The cells were centrifuged at 3000× *g* for 5 min. The concentration of glucose in the supernatant was determined according to the reported methods [29]. The pellets were washed with ddH_2_O twice and filtered through pre-dried filter papers (Whatman GF/C, GE Healthcare Bio-Sciences, Pittsburgh, PA, USA). Then the cells on the discs were dried to constant weight in a vacuum oven at 80 °C.

### 4.6. Statistical Analysis

All data were obtained from three repetitions and analyzed by using the one-way analysis of variance (ANOVA) with subsequent post hoc multiple comparison by least-significant difference (LSD) test, which were calculated by the software of IBM SPSS Statistics 20 (IBM Corporation, Armonk, NY, USA). Statistically significant values were defined as *p* < 0.05.

## 5. Conclusions

In conclusion, a high EPS production and stable mutant of *C. cohnii* was obtained by using the novel mutation technique of ARTP. Results of the present work supported that ARTP has the potential to enhance EPS yield of microalgae as well as other similar microorganisms. Further studies are needed to figure out the potential biological activities of EPS from the *C. cohnii* mutants.

## References

[1] Boddohi S., Kipper M.J. (2010). Engineering nanoassemblies of polysaccharides. Adv. Mater..

[2] Laurienzo P. (2010). Marine polysaccharides in pharmaceutical applications: An overview. Mar. Drugs.

[3] Wu J.Y., Chen X., Siu K.C. (2014). Isolation and structure characterization of an antioxidative glycopeptide from mycelial culture broth of a medicinal fungus. Int. J. Mol. Sci..

[4] Yao Y., Shi Z.X., Ren G.X. (2014). Antioxidant and immunoregulatory activity of polysaccharides from quinoa (*Chenopodium quinoa* Willd.). Int. J. Mol. Sci..

[5] Sun L.Q., Wang L., Li J., Liu H.H. (2014). Characterization and antioxidant activities of degraded polysaccharides from two marine Chrysophyta. Food Chem..

[6] Guzman S., Gato A., Lamela M., Freire-Garabal M., Calleja J.M. (2003). Anti-inflammatory and immunomodulatory activities of polysaccharide from *Chlorella stigmatophora* and *Phaeodactylum*
*tricomutum*. Phytother. Res..

[7] Samarakoon K.W., Ko J.Y., Shah M.M.R., Lee J.H., Kang M.C., O-Nam K., Lee J.B., Jeon Y.J. (2013). *In vitro* studies of anti-inflammatory and anticancer activities of organic solvent extracts from cultured marine microalgae. Algae.

[8] Gardeva E., Toshkova R., Yossifova L., Minkova K., Gigova L. (2012). Cytotoxic and apoptogenic potential of red microalgal polysaccharides. Biotechnol. Biotechnol. Equip..

[9] Raposo M.F.D., de Morais A., de Morais R. (2014). Influence of sulphate on the composition and antibacterial and antiviral properties of the exopolysaccharide from *Porphyridium cruentum*. Life Sci..

[10] Kim M., Yim J.H., Kim S.Y., Kim H.S., Lee W.G., Kim S.J., Kang P.S., Lee C.K. (2012). *In vitro* inhibition of influenza A virus infection by marine microalga-derived sulfated polysaccharide p-KG03. Antivir. Res..

[11] Raposo M.F.D., de Morais R., de Morais A. (2013). Health applications of bioactive compounds from marine microalgae. Life Sci..

[12] Pignolet O., Jubeau S., Vaca-Garcia C., Michaud P. (2013). Highly valuable microalgae: Biochemical and topological aspects. J. Ind. Microbiol. Biotechnol..

[13] Dewapriya P., Kim S.-K. (2014). Marine microorganisms: An emerging avenue in modern nutraceuticals and functional foods. Food Res. Int..

[14] Zhang X., Zhang X.F., Li H.P., Wang L.Y., Zhang C., Xing X.H., Bao C.Y. (2014). Atmospheric and room temperature plasma (ARTP) as a new powerful mutagenesis tool. Appl. Microbiol. Biotechnol..

[15] Fang M.Y., Jin L.H., Zhang C., Tan Y.Y., Jiang P.X., Ge N., Li H.P., Xing X.H. (2013). Rapid mutation of *Spirulina platensis* by a new mutagenesis system of atmospheric and room temperature plasmas (ARTP) and generation of a mutant library with diverse phenotypes. PLoS ONE.

[16] Wang Q., Feng L.R., Wei L., Li H.G., Wang L., Zhou Y., Yu X.B. (2014). Mutation breeding of lycopene-producing strain *Blakeslea trispora* by a novel atmospheric and room temperature plasma (ARTP). Appl. Biochem. Biotechnol..

[17] Chang K.J.L., Nichols C.M., Blackburn S.I., Dunstan G.A., Koutoulis A., Nichols P.D. (2014). Comparison of thraustochytrids *Aurantiochytrium* sp., *Schizochytrium* sp., *Thraustochytrium* sp., and *Ulkenia* sp. for production of biodiesel, long-chain omega-3 oils, and exopolysaccharide. Mar. Biotechnol..

[18] De Swaaf M.E., Grobben G.J., Eggink G., de Rijk T.C., van der Meer P., Sijtsma L. (2001). Characterisation of extracellular polysaccharides produced by *Crypthecodinium cohnii*. Appl. Microbiol. Biotechnol..

[19] Mohamed S., Hashim S.N., Rahman H.A. (2012). Seaweeds: A sustainable functional food for complementary and alternative therapy. Trends Food Sci. Technol..

[20] Mendes A., Reis A., Vasconcelos R., Guerra P., da Silva T.L. (2009). *Crypthecodinium cohnii* with emphasis on DHA production: A review. J. Appl. Phycol..

[21] Hazen K.C. (2013). Influence of DMSO on antifungal activity during susceptibility testing *in vitro*. Diagn. Microbiol. Infect. Dis..

[22] Laroussi M., Lu X. (2005). Room-temperature atmospheric pressure plasma plume for biomedical applications. Appl. Phys. Lett..

[23] Leduc M., Guay D., Coulombe S., Leask R.L. (2010). Effects of non-thermal plasmas on DNA and mammalian cells. Plasma Process. Polym..

[24] Lu Y., Wang L.Y., Ma K., Li G., Zhang C., Zhao H.X., Lai Q.H., Li H.P., Xing X.H. (2011). Characteristics of hydrogen production of an *Enterobacter aerogenes* mutant generated by a new atmospheric and room temperature plasma (ARTP). Biochem. Eng. J..

[25] Degeest B., de Vuyst L. (2000). Correlation of activities of the enzymes alpha-phosphoglucomutase, UDP-galactose 4-epimerase, and UDP-glucose pyrophosphorylase with exopolysaccharide biosynthesis by *Streptococcus thermophilus* LY03. Appl. Environ. Microbiol..

[26] Xu J.W., Ji S.L., Li H.J., Zhou J.S., Duan Y.Q., Dang L.Z., Mo M.H. (2015). Increased polysaccharide production and biosynthetic gene expressions in a submerged culture of *Ganoderma lucidum* by the overexpression of the homologous alpha-phosphoglucomutase gene. Bioprocess Biosyst. Eng..

[27] Grobben G., van Casteren W., Schols H., Oosterveld A., Sala G., Smith M., Sikkema J., de Bont J. (1997). Analysis of the exopolysaccharides produced by *Lactobacillus delbrueckii* subsp. bulgaricus NCFB 2772 grown in continuous culture on glucose and fructose. Appl. Microbiol. Biotechnol..

[28] Dai J., Zhu S., Tang J., Wang M., Yin H.P., Chen S.W. (2007). Analysis of monosaccharide composition in polysaccharides from *D. salina* by pre-column derivatization high performance liquid chromtography. J. Instrum. Anal..

[29] Miller G.L. (1959). Use of dinitrosalicylic acid reagent for determination of reducing sugar. Anal. Chem..

