# Correction: Liu et al. Mutation Breeding of Extracellular Polysaccharide-Producing Microalga *Crypthecodinium cohnii* by a Novel Mutagenesis with Atmospheric and Room Temperature Plasma. *Int. J. Mol. Sci.* 2015, *16*, 8201–8212

**DOI:** 10.3390/ijms26146756

**Published:** 2025-07-15

**Authors:** Bin Liu, Zheng Sun, Xiaonian Ma, Bo Yang, Yue Jiang, Dong Wei, Feng Chen

**Affiliations:** 1 School of Light Industry and Food Sciences, South China University of Technology, Guangzhou 510641, Chinafewd304@scut.edu.cn (D.W.); 2 Institute for Food and Bioresource Engineering, College of Engineering, Peking University, Beijing 100871, China; zsun@shou.edu.cn (Z.S.); ydchmn@126.com (X.M.);; 3 College of Fisheries and Life Science, Shanghai Ocean University, Shanghai 201306, China; 4 School of Food Science, Jiangnan University, Wuxi 214122, China; jiangyue@tust.edu.cn; 5 Singapore-Peking University Research Centre for a Sustainable Low-Carbon Future, CREATE Tower, Singapore 138602, Singapore

In the original publication [1], the corresponding author has been changed from Feng Chen to Bin Liu, due to the passing of the original corresponding author, Feng Chen, the corresponding authorship has been changed to the first author. And there was a mistake in Figure 3 as published. The authors put the wrong picture of the mutant M9 in Figure 3. The corrected Figure 3 appears below. The authors state that the scientific conclusions are unaffected. This correction was approved by the Academic Editor. The original publication has also been updated.

## Figures and Tables

**Figure 3:**
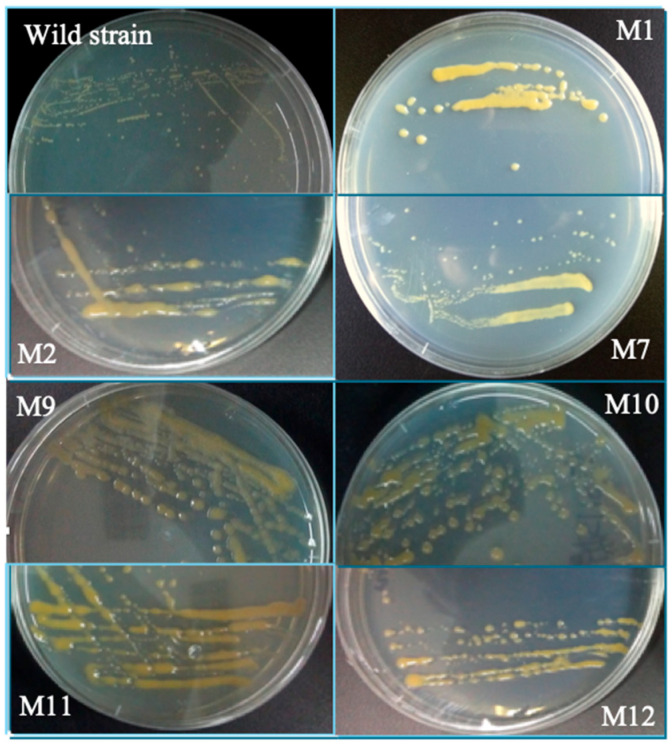
Colonies of wild type of *C. cohnii* and the mutants: the wild type and selected mutants of *C. cohnii* were grown on solid agar plates and incubated for 14 days.
